# Leveraging computational tools to combat malaria: assessment and development of new therapeutics

**DOI:** 10.1186/s13321-024-00842-z

**Published:** 2024-05-02

**Authors:** Nomagugu B. Ncube, Matshawandile Tukulula, Krishna G. Govender

**Affiliations:** 1 School of Chemistry and Physics, College of Agriculture, Engineering and Science (CAES), University of KwaZulu-Natal, Westville Campus, Durban, 4001 South Africa; 2 Department of Chemical Sciences, University of Johannesburg, Johannesburg, 2028 South Africa

**Keywords:** Computer-aided drug design, in silico techniques, Machine learning, AI, QSAR

## Abstract

**Abstract:**

As the world grapples with the relentless challenges posed by diseases like malaria, the advent of sophisticated computational tools has emerged as a beacon of hope in the quest for effective treatments. In this study we delve into the strategies behind computational tools encompassing virtual screening, molecular docking, artificial intelligence (AI), and machine learning (ML). We assess their effectiveness and contribution to the progress of malaria treatment. The convergence of these computational strategies, coupled with the ever-increasing power of computing systems, has ushered in a new era of drug discovery, holding immense promise for the eradication of malaria.

**Scientific contribution:**

Computational tools remain pivotal in drug design and development. They provide a platform for researchers to explore various treatment options and save both time and money in the drug development pipeline. It is imperative to assess computational techniques and monitor their effectiveness in disease control. In this study we examine renown computational tools that have been employed in the battle against malaria, the benefits and challenges these tools have presented, and the potential they hold in the future eradication of the disease.

## Introduction

Malaria remains a disease of concern to the health community. The latest World Health Organization (WHO) report indicates that there were approximately 247 million malaria cases in 2021, an increase from the 245 million cases reported in 2020, with most of this increase originating from African countries [1]. The discovery of new drugs for malaria is becoming exceptionally difficult due to the constant emergence of resistance that outpaces the development of new medicaments. The combination of artemisinin and its derivatives is slowly becoming redundant due to resistant strains to partner drugs [2]. Plasmodium parasite species responsible for infecting humans are *Plasmodium falciparum (P. falciparum)*, *Plasmodium vivax (P. vivax)*, *Plasmodium ovale (P. ovale)*, *Plasmodium malariae (P. malariae)*, and *Plasmodium knowlesi (P. knowlesi)* [3]. Mortality from malaria is increasing at an alarming rate despite the efforts to eradicate the parasites. In 2020, there was a 10% increase in mortality cases, with 63 000 deaths reported between 2019 and 2021, mainly because of disrupted essential malaria services due to the COVID-19 pandemic [1].

**Fig. 1:**
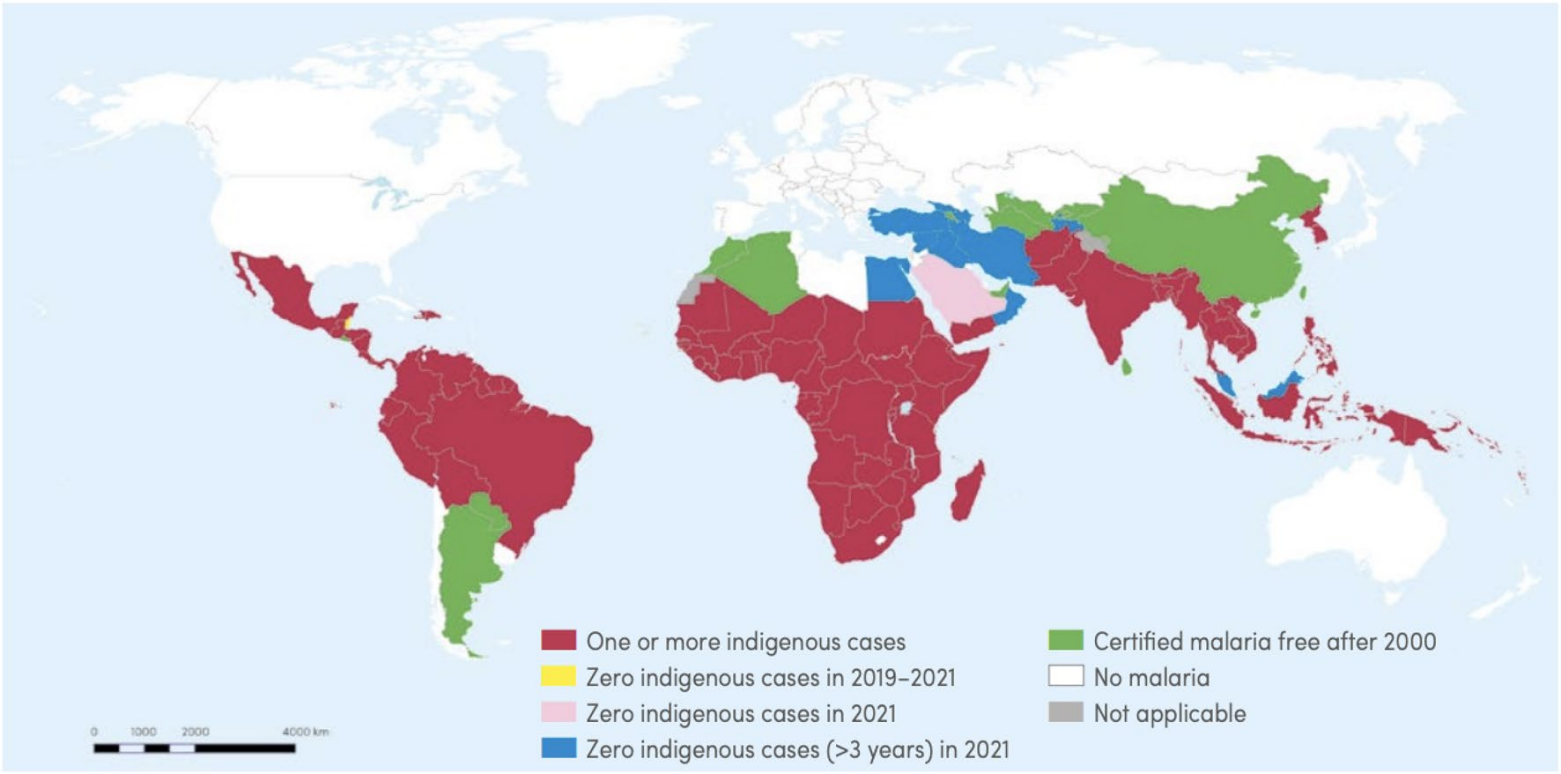
Geographical distribution of malaria [1]

**Fig. 2:**
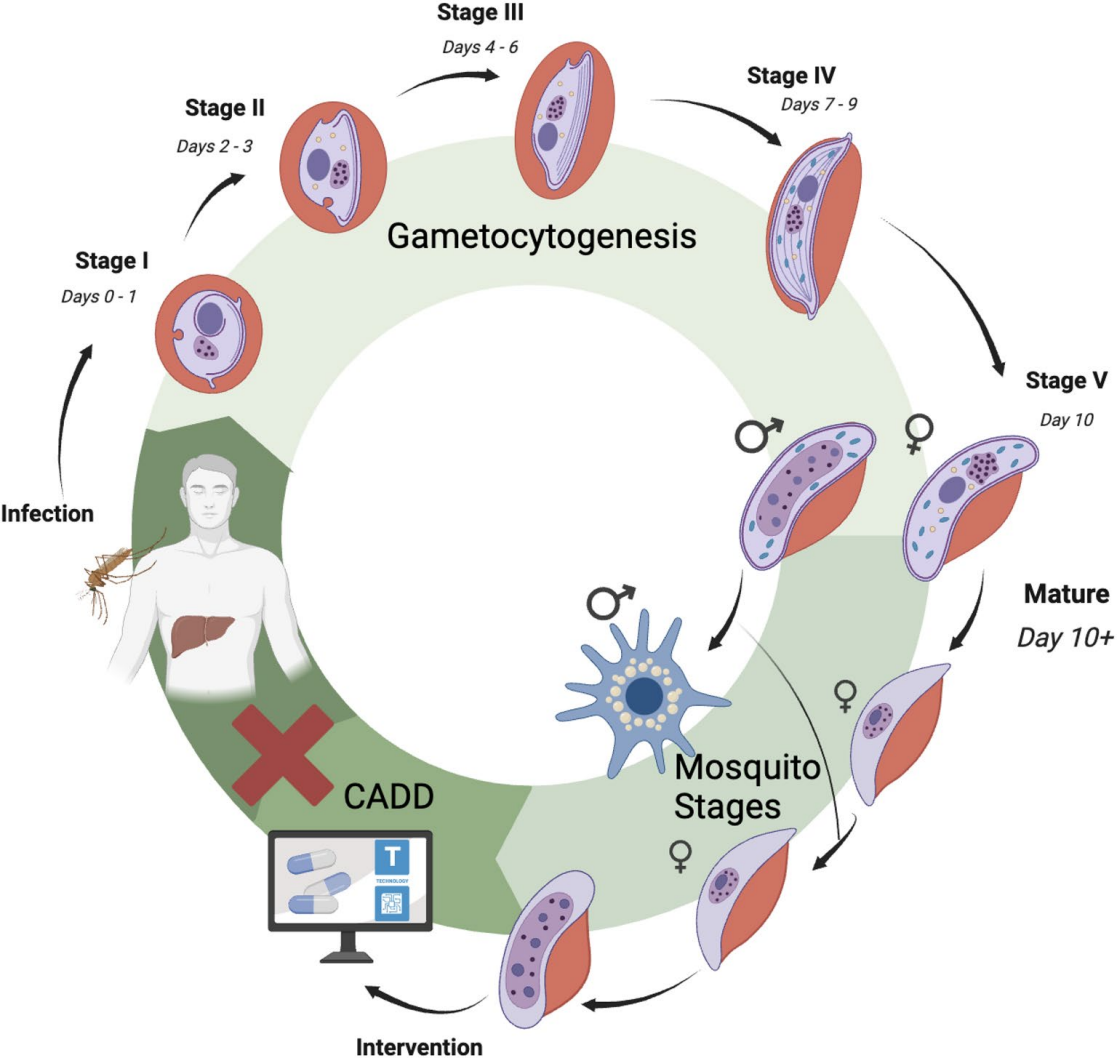
Life cycle of *Plasmodium falciparum* and proposed intervention

Figure 1 shows the geographical distribution of malaria worldwide, with Africa, Asia, and the Americas being the hardest hit. In most cases, malaria spreads through bites of anopheles mosquitoes. However, infected blood and congenital transmission cases have been reported from time to time [4, 5]. The malaria parasite’s lifecycle begins in the mosquito, then develops in the host cells and infection begins (Fig. 2). Various stages in this lifecycle are vital to the parasite’s survival and offer clear-cut targets for chemotherapy development. For instance, computer-aided drug discovery (CADD) approaches aim to inhibit the progression of parasite development so that the host does not get infected by the vector that carries the parasite.

Most computational efforts to develop new antimalarial drugs have targeted modifying existing molecules’ scaffolding to compensate for the resistance [6]. This approach screens a library of potential compound actives for activity against the parasite in phenotypic, whole-cell targets or proteins, which are typically crucial for parasitic survival. Once a superior scaffolding is selected, the proposed compounds are synthesized and tested against whole parasites or specific proteins in tests that reveal a structure–activity relationship (SAR), which can predict the effects of chemical modifications on the molecule. Flannery and coworkers claim that SAR can reveal the effects and impact of chemical modifications and ultimately, compound identification [7].

## Effectiveness of drugs designed using computational tools

### Molecular docking

**Fig. 3:**
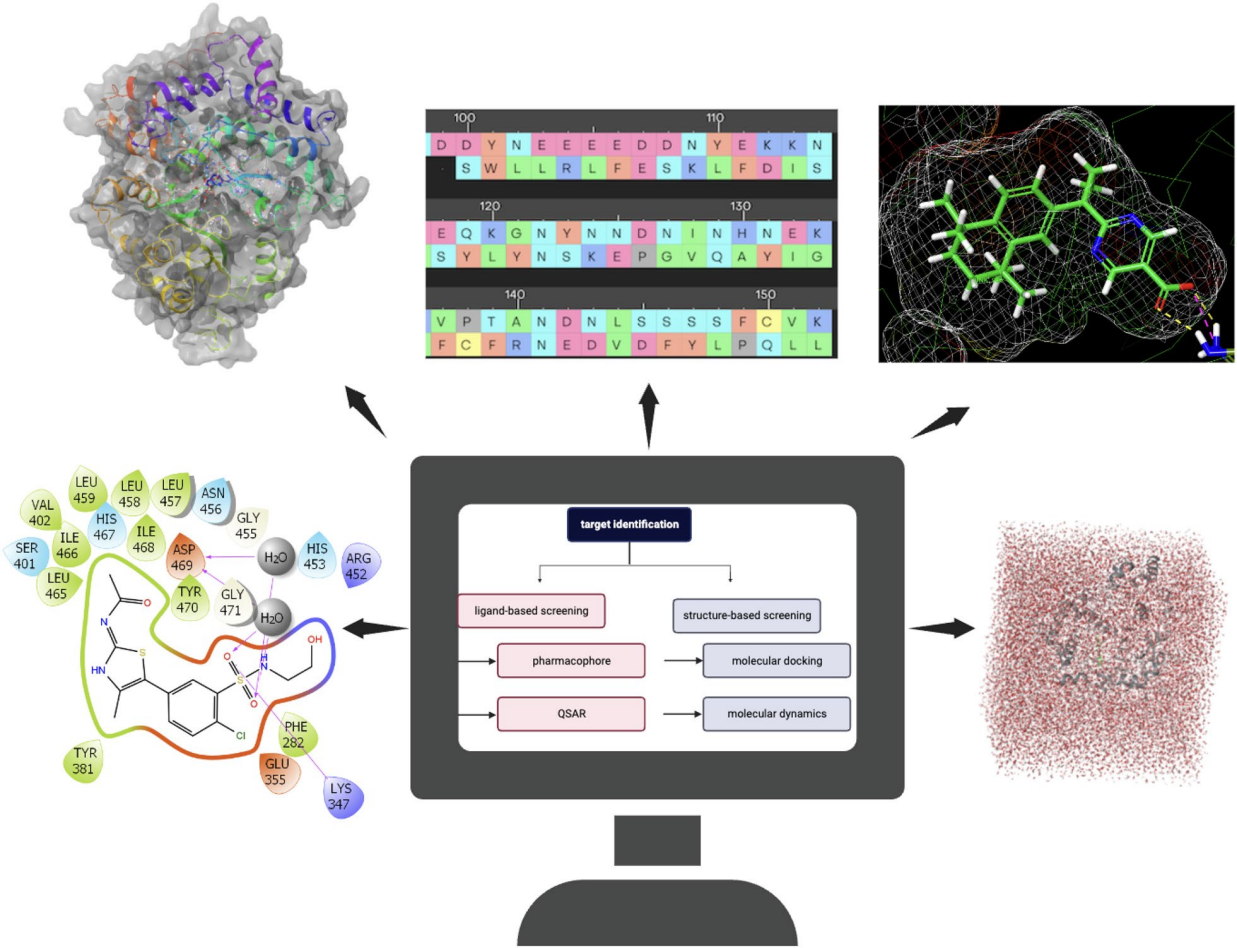
A summary of common computational techniques employed in drug discovery

Computational chemistry dates back to 1928 when physicists attempted to solve Schrodinger’s equation using machines [8]. Today, it is a valuable precursor in the drug design and pharmaceutical industry as it is economical, time-saving, and unlimited regarding the chemical space one can explore [9]. Figure 3 below summarizes a few computational/in silico techniques in the drug discovery field.

One in silico technique used in the computational discovery of drugs is the molecular docking approach. According to Pinzi and colleagues, docking approaches facilitate the identification of novel compounds for therapeutic analysis [10]. Saikia et al., in turn, describe molecular docking as the process where small molecules are docked into macromolecular structures for scoring the complementary values at the binding sites [11]. This technique allows the prediction of ligand-target interactions at the molecular level as well as SAR delineation.

Bhagat et al. also reiterates how molecular docking applies the scoring function for predicting the binding affinity of ligands and proteins. Its drug-receptor interactions predict the molecule’s affinity and activity. [12]Stanzione et al. further details the increasing importance of molecular docking in the drug discovery process, especially when combined with other computational approaches such as virtual screening [13]. Chaudhary and Mishra further add to the discussions by asserting that molecular docking predicts the favored ligand orientation against the receptor to produce a stable complex [14]. It is applied to predict a drug candidate’s binding orientation against its protein targets and subsequent potential affinity and activity. Molecular docking is a renowned technique that has been constantly used to identify new ligands [15]. It has been extensively used in the ongoing search for new drugs for malaria and other diseases of concern to the global health community [16].

### Artificial intelligence method

A book chapter by Ghosh and Choudhuri in 2021 addresses how artificial intelligence (AI) technology has been applied to design new malarial drugs [17]. This approach is considered a more time-saving and cost-reducing approach compared to the classical drug discovery models. In Ghosh and Choudhuri’s text, reference is made to the use of machine learning or other computational methods, specifically to the deep learning-based technique (DeepMalaria) designed by Arashadi et al. [17, 18] This approach uses a graph-based model and SMILES (Simplified Molecular Input Line Entry System) for predicting potential antimalarial compounds.

The in silico techniques for analysis of anti-plasmodium compounds include the protein–protein interaction network of *P. falciparum* and the human host. This was developed by Monika Samant and colleagues through the integration of experimental data and computational prediction, where the interolog method is used to predict interactions [19]. There is further emphasis on how genomics data is used for drug target selection, with the first step of identifying a potential target for the drug discovery project. Hence, for target identification, transcriptomics data can be utilized effectively, with differential gene expression analysis providing information about the variance in gene expression between normal and diseased cells [17]. According to this article, AI primary drug screening entails image processing, sorting, and classifying cells.

Similarly, Arshadi et al. document the utilization of structure-based or ligand-based models in AI for highly accurate chemical property prediction [18]. In this case, AI applies the learning of patterns within the data and searches for hit compounds more effectively than blind-search high-throughput screening (HTS). Arshadi et al. in 2020 [18], like Ghosh and Choudhury in 2021 [17], refer to the DeepMalaria deep-learning process that can use SMILES to predict anti-*P. falciparum* inhibitory properties of compounds.

The approach uses the GlaxoSmithKline (GSK) library of compounds to source for antiplasmodial hit compounds currently used in finding novel drug candidates for malaria. AI is critical for biomedical research, considering the available large datasets such as those of whole genome sequencing. Its application will be in the form of ligand-based virtual screening (VS), structure-based VS, target prediction, metabolomics approaches, or de novo molecular design. Hence, this article establishes the accuracy of DeepMalaria, where eight potentially repurposable compounds are predicted as potential antimalarials [20] based on deep learning that work at all stages of *P. falciparum* growth. These potentially repurposable compounds are Azidothromycin, Cyclosporin A, Esomeprazole, Pentamiine, Omeprazole, Auranofin, Loperamide and amlodipine [18].

Another article that discusses machine learning as an antimalarial drug-discovery method is Ashdown et al. [21]. In this text, the researchers note that machine learning methods can be alternatives to manual image analysis as they can use deep neural networks (DNNs) to learn and represent data [21]. Supervised ML has already been applied to classify imaging data based on binning inputs into discrete outputs defined by humans. In their study, the researchers used ML to images of asynchronous *P. falciparum* cultures. In a similar argument as Oguike et al. in 2022 and Lin et al. in 2020, Ashdown and colleagues concluded that the ML model could identify effective drugs and use life cycle stage and morphological outliers to cluster the identified drugs [22, 23].

### Quantitative structure–activity relationship (QSAR) modeling

**Fig. 4:**
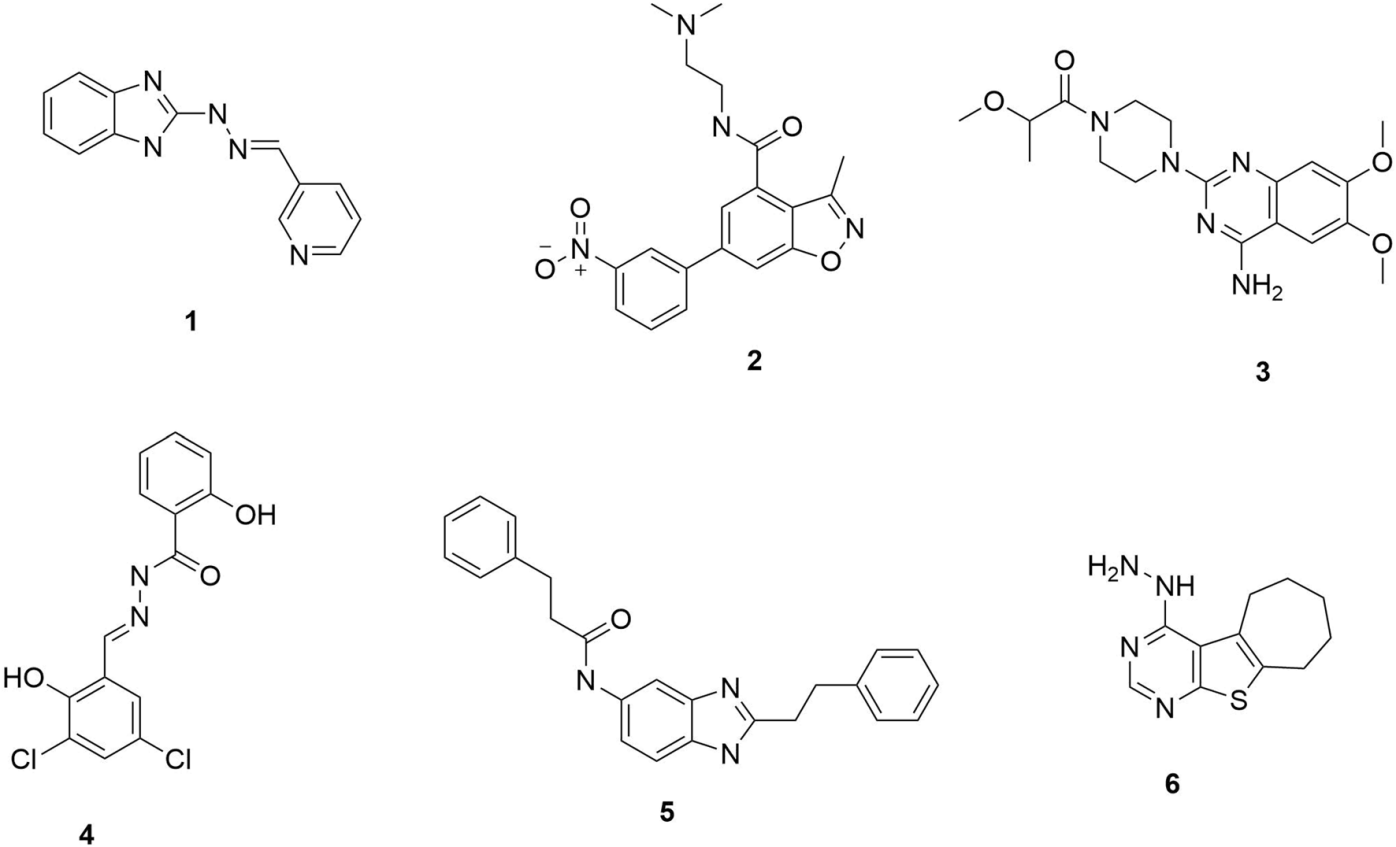
Six of the leading 21 anti-haemozoin compounds reported in Nguyen et al. study [24]

Several researchers have documented the application of quantitative structure–activity Relationship (QSAR) modeling for antimalarial drug discovery, and these include Nguyen et al. [24], Yousefinejad et al. [25], Lima and colleagues [26], Hou et al. [27], and Hadni and Elhallaoui [28]. In Nguyen et al., there is a discussion of 2D-QSAR modeling, an in silico technique that can reduce the time and cost of the drug discovery process, just like AI (Oguike et al., 2022) [22]. This method is applied in rational drug design through its predictive model that provides a mathematical correlation between the compounds’ structural properties and their antimalarial activities. A reliable QSAR model enables the prediction of the biological activities of molecules using molecular descriptors in methodologies like multiple linear regression, artificial neural networks, partial least squares, and heuristic methods. The study by Nguyen et al. used a 2D-QSAR model based on a database of 21 anti-haemozoin compounds and studied the structure–activity relationship of the haemozoin inhibitors as antimalarial agents. Figure 4 shows six (**1**–**6**) of the best compounds among the 21 in terms of anti-haemozoin activity.

**Fig. 5:**
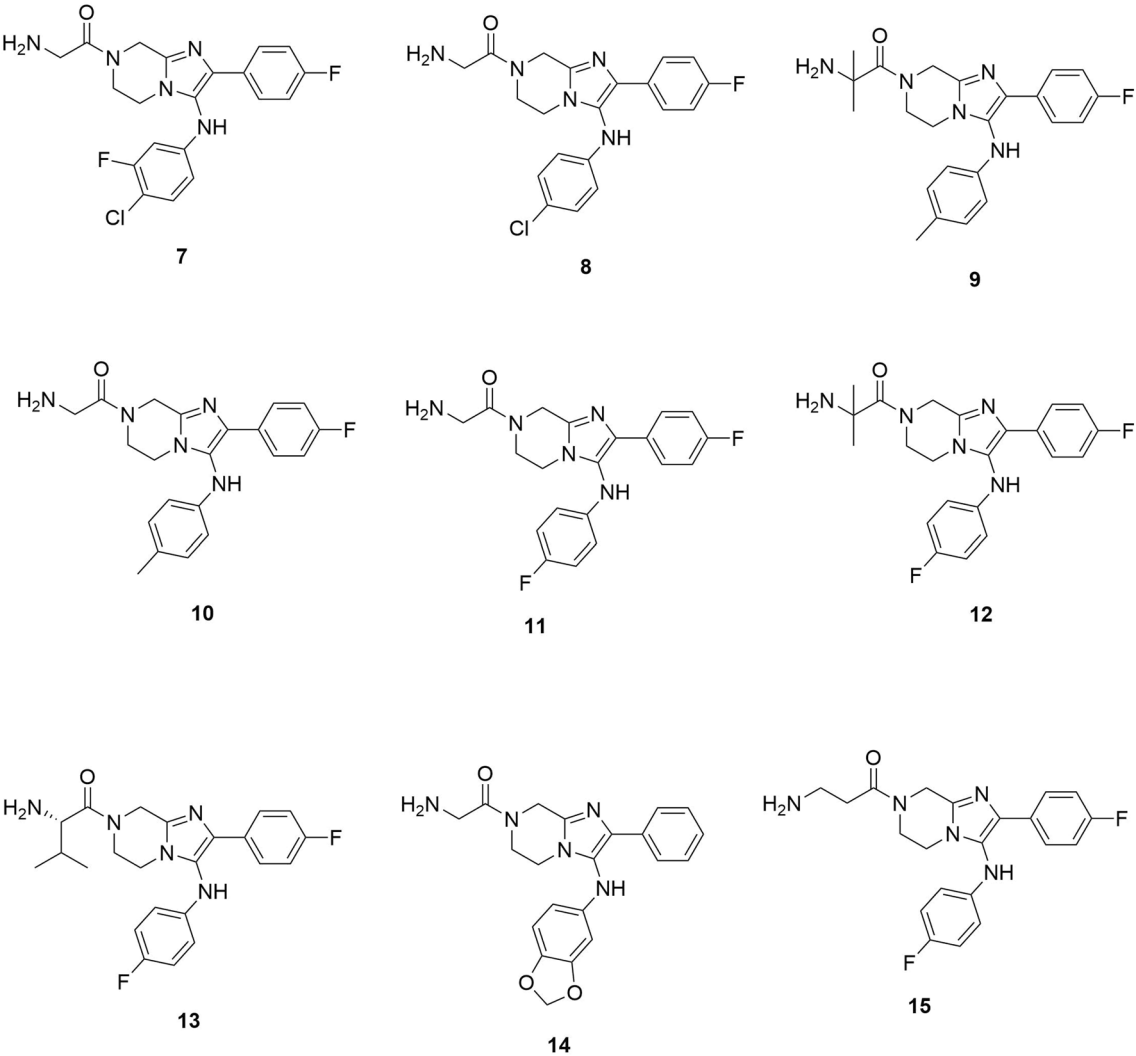
Some of the most active imidazopiperazine compounds reported in Yousefinejad et al. study [25]

**Fig. 6:**
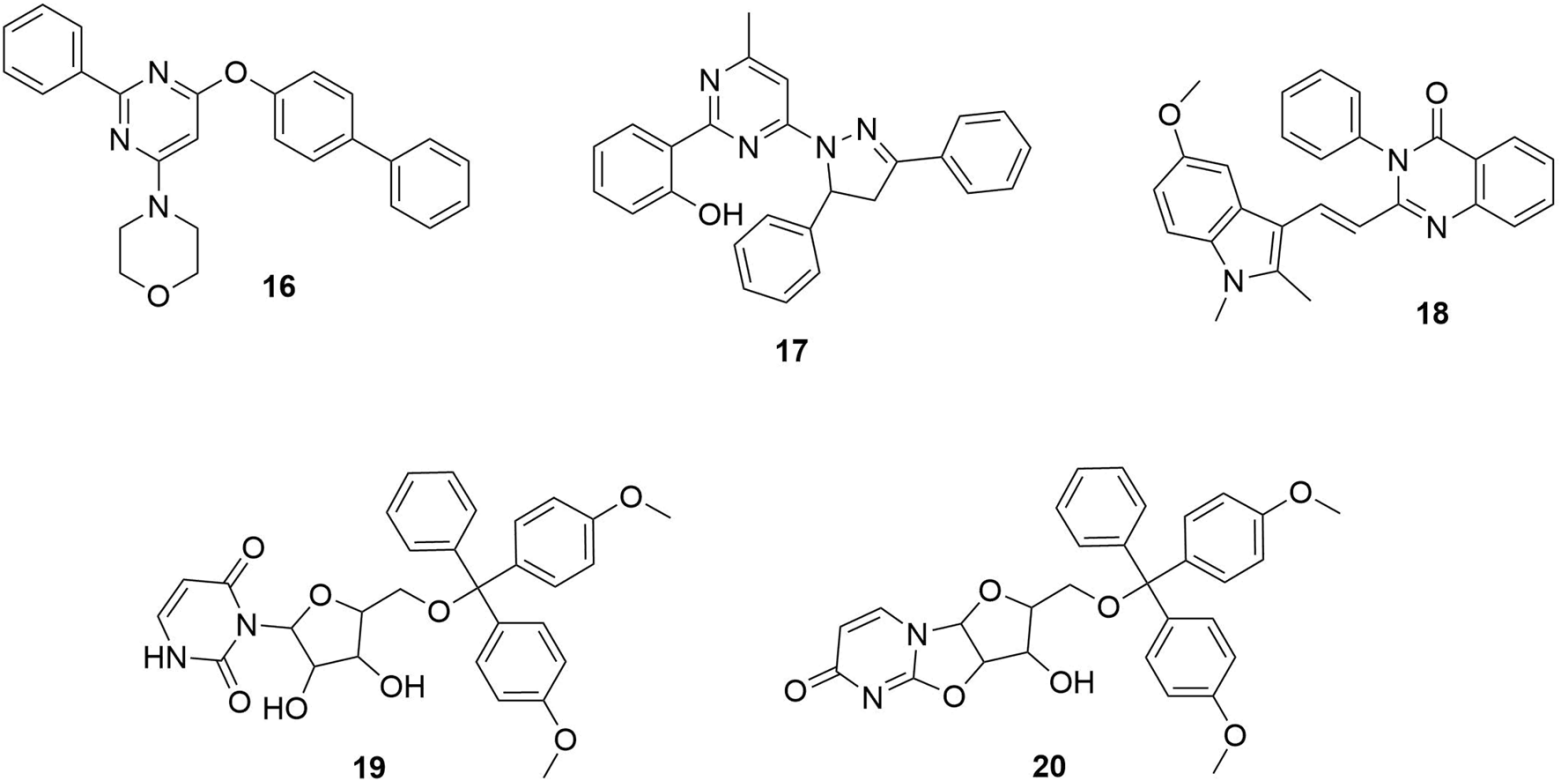
Five virtual hit compounds discovered from the ChemBridge library [26]

In Yousefinejad et al. [25] study, the researchers did a QSAR on the antimalarial activity of imidazolopiperazine compounds (Fig. 5 shows some of the most active compounds, **7**–**15**, from this family) based on artificial neural networks (ANN). The results of Yousefinejad et al. [25] showed that QSAR models, which highlight structure–activity relationships associated with structural features of compounds that possess antimalarial activities, are suited for designing and modifying antimalarial compounds(Lima et al. [26] also used a combi-QSAR approach with the virtual screening of the ChemBridge’s Hit2Lead library[29] to discover five virtual hits (**16**–**20**) (Fig. 6) with potency against *P. falciparum* and *P. berghei*). The experiment evaluated the potential of *P. falciparum* Deoxyuridine 5′-triphosphate nucleotido-hydrolase (*Pf*dUTPase) inhibitors as antimalarial agents. Their findings indicate that merging 2D- and 3D-QSAR models allowed the selection of new potential selective *Pf*dUTPase inhibitors. These can be potent alternatives for antimalarial drug combinations.

**Fig. 7:**
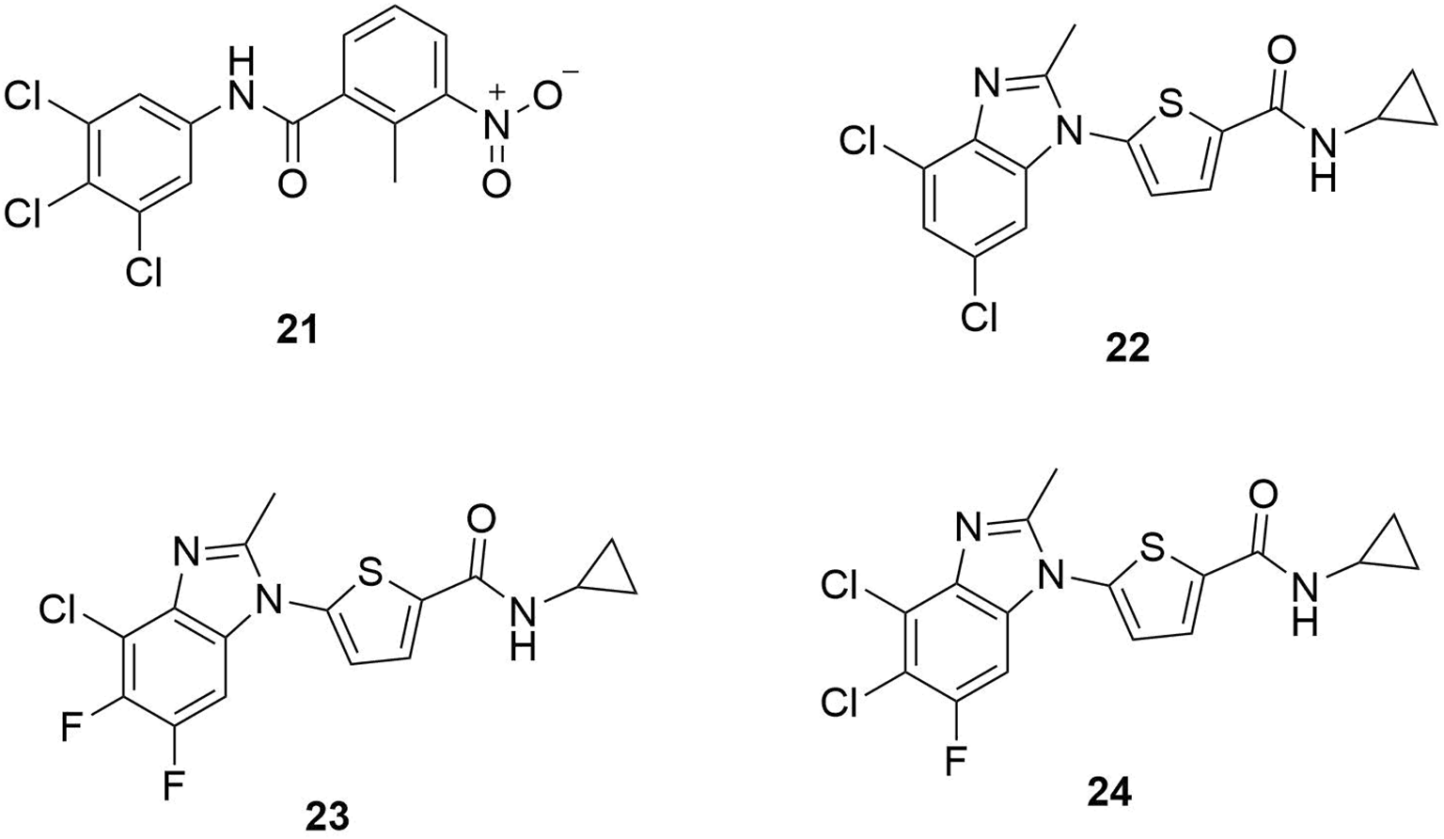
The best four compounds from the study reported by Hou et al.[27]

It was also found by Hou et al. [27] that QSAR models that predict *Pf*DHODH inhibitors’ bioactivity have a good prediction ability and can be used to discover the factors affecting antimalarial activity of these inhibitors and for further drug development. Figure 7 shows the best four compounds, **21**–**24**, found through QSAR models developed by Hou et al.

## Main challenges and benefits in the development of new inhibitors

### Benefits

According to Sliwoski et al., computer-aided drug design (CADD) has been influential in the therapeutic development of small molecules [30]. The methods are either structure- or ligand-based. Hasan et al. assert that computational models of drug discovery are beneficial in addressing the time-consuming and expensive challenges related to drug research and development [31]. Sliwoski et al. [30] emphasizes that with CADD, it is possible to increase the hit rate of novel drug compounds since computational models use more targeted search compared to traditional high throughput screening. CADD explains both the molecular basis of therapeutic activity and predicts potential derivatives with improved activity.

Computational models also facilitate target validation, which demonstrates the active engagement of a cellular target in a disease state and that modulation is potentially therapeutic [31]. As indicated in Adelusi et al. [32], drug discovery techniques using molecular modeling further accelerate drug discovery, with researchers and organizations using the components of molecular docking, absorption, distribution, metabolism, elimination and toxicity (ADMET) modeling, and molecular dynamics [32]. These mechanisms come with the advantages associated with determining drug efficacy or potency, with ADMET modeling specifically able to dictate the clinical success of drugs during the clinical trials phase.

Sadiku et al. make another crucial point regarding how computational pharmacology addresses the limitations of direct experimentation since this model can be used to study complex natural phenomena that would otherwise be too dangerous [33]. The method is also useful where practical and ethical considerations can limit experimentation with real systems. Further, in-silico methods can provide insight into the toxicity of drug compounds [34]. Therefore, the review of the benefits of computational models indicates their value in reducing the costs of drug discovery and validating target compounds for synthesizing antimalarials.

### Molecular docking

In applying molecular docking for CADD in medicinal chemistry, its benefits have been observed as including the effectiveness, quickness, and low-cost nature of the technique when applied in scientific and corporate contexts [35]. The uses of docking in drug discovery are helping to rationalize ligands activity towards a target as part of performing structure-based drug design (SBDD). While also noting that docking reveals the novel compound of interest in therapeutic design, as well as forecasting the ligand–protein interaction while further delineating the SAR. Sethi et al. agree by highlighting that molecular docking has been part of rationalizing the drug discovery path [36]. This is especially because docking can be integrated with other classical techniques to discover drugs. Arjmand et al. article further documents the usefulness of docking as part of the methods for designing cell-targeting therapeutics [37]. Structure-based molecular docking has also been found to be capable of prioritizing among ultra-large libraries, and this is crucial in finding unusually potent and selective molecules [38]. This works at the molecular level and as libraries grow larger, docking results will keep improving.

## Challenges

In silico models of drug discovery have their limitations besides their strengths. Although in silico methods offer more practical and economical experiments thus proving valuable, they do present a few challenges. They limit the use of animal models which aid in the design of safe drug candidates. Another challenge is that in silico methods often need experimental validation as they cannot be solely trusted without validation. For instance, in Sacan et al., the researchers claim that determining the protein structure is critical in computational drug discovery, as models are only used to represent the underlying physical structure [39]. Consequently, there are limitations in resolving the experimental data collected, biases and errors in the knowledge base, and the likelihood of incomplete optimization methods that can influence the protein structure quality. This is why new and improved AI models are available to try to better predict poor-quality protein structures and why things like homology modeling are an effective alternative.

The use of AI to facilitate drug discovery is proving efficient. In 2023, latest developments in AI included virtual clinical trials thus reducing regulatory compliance issues. Some companies such as Protai in Israel are developing an AI driven drug discovery platform. Other companies such as Netabolics are predicting the effects of new drugs by digitising human cells. There is significant progress in the use of AI to facilitate the drug discovery process [40].

## How computational tools have helped curb malaria

### Molecular docking

**Fig. 8:**
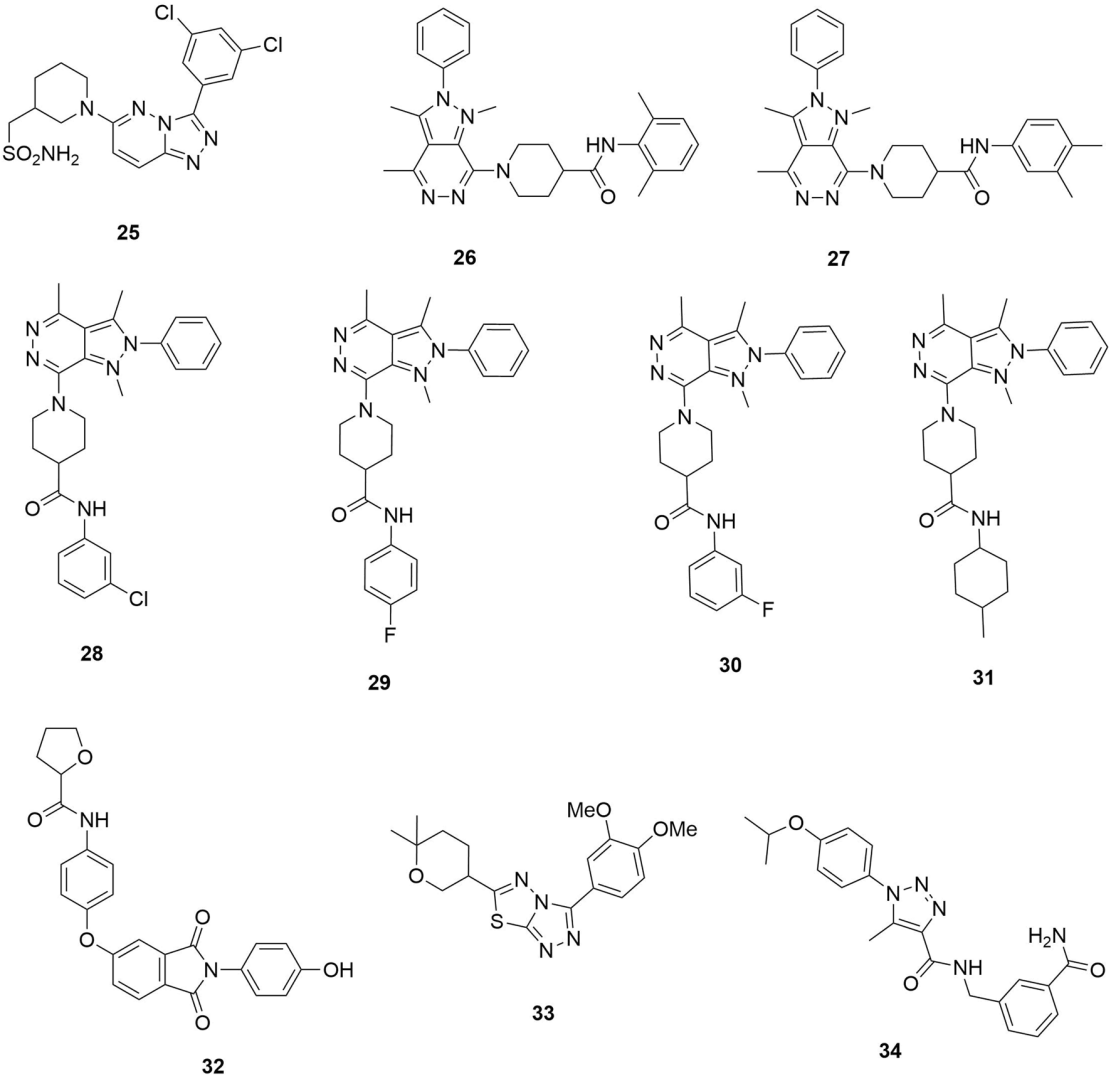
Novel inhibitors of *Pf*PI4KIIIβ by Ibrahim et al. [41]

Molecular docking has been applied in the discovery and design of antimalarial drugs. In Ibrahim et al., the study identified how the inhibition of *P. falciparum* phosphatidylinositol beta 4 kinase (*Pf*PI4KIIIβ) is considered a promising therapy for treating malaria infection [41, 42]. *Pf*PI4KIIIβ inhibition is considered as preventing membrane ingression when the plasmodium lifecycle is completing the asexual erythrocytic stage. Hence, Ibrahim et al. [41] used molecular docking to construct and validate the *Pf*PI4KIIIβ comparative model through a ligand- and structure-based drug discovery method meant to identify novel, potent, and selective inhibitors (**25**–**34**) of *Pf*PI4KIIIβ to act as antimalarial agents (Fig. 8).

From the results of the study, Ibrahim et al. (2020) first noted that docking allows the construction of a model of *Pf*PI4KIIIβ based on the PI4KIIIβ X-ray resolved structure via homology modeling due to the absence of a crystal structure of *Pf*PI4KIIIβ.

Rajkhowa et al. have performed a similar study to Ibrahim et al. [41] whereby they noted that the phosphatidylinositol-4-OH kinase (PI(4)K) type IIIβ, a lipid kinase, is a target for imidazopyrazines as rapid resistance to antimalarial drugs persists [43]. Imidazopyrazines are categorized as antimalarial compounds inhibiting the intracellular development of various *Plasmodium* species across infection stages in the vertebrate host [43]. Ncube et al. [44] recently undertook an in-depth analysis of the binding pocket of *Pf*PI4KIIIβ and discovered that Lys 347, Val 396 amino acid residues play a critical role in binding to known *Pf*PI4KIIIβ inhibitors [44].

The findings of this research identified ten molecules with good binding characteristics from docking studies. Therefore, the modeling of the *Pf*PI4KIIIβ active site shows potential for developing highly specific inhibitors that are potential antimalarial therapies.

**Fig. 9:**
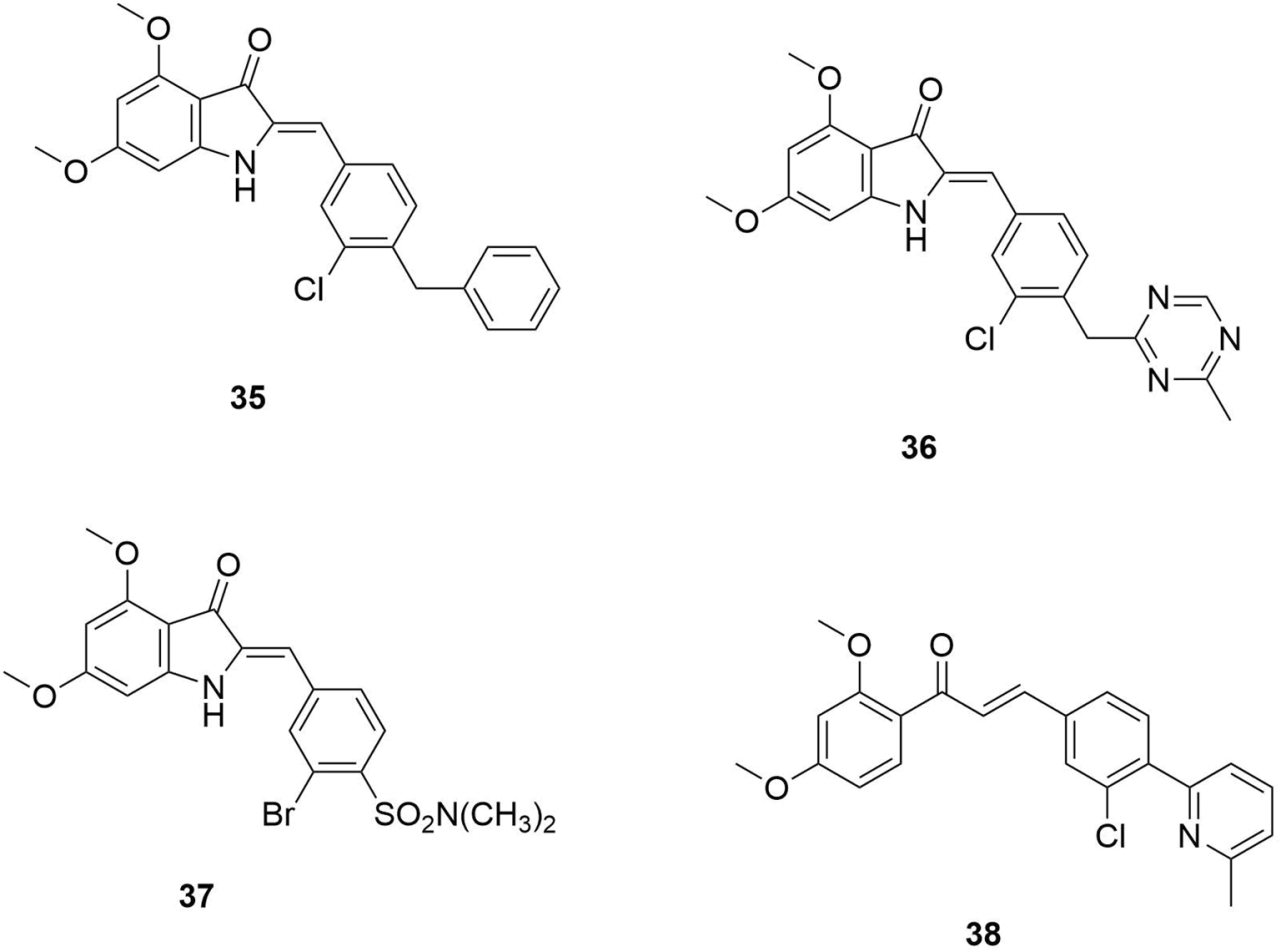
Ligands that have undergone 3D QSAR in order to improve their function [28]

Docking has also been applied in identifying *P.falciparum*-dihydrofolate reductase (*Pf*DHFR) inhibitors (**35**–**38**), as indicated by Hadni and Elhallaoui (Fig. 9) [28]. *Pf*DHFR enzyme is responsible for producing folates required for DNA synthesis in the malaria parasite [45].

**Fig. 10:**
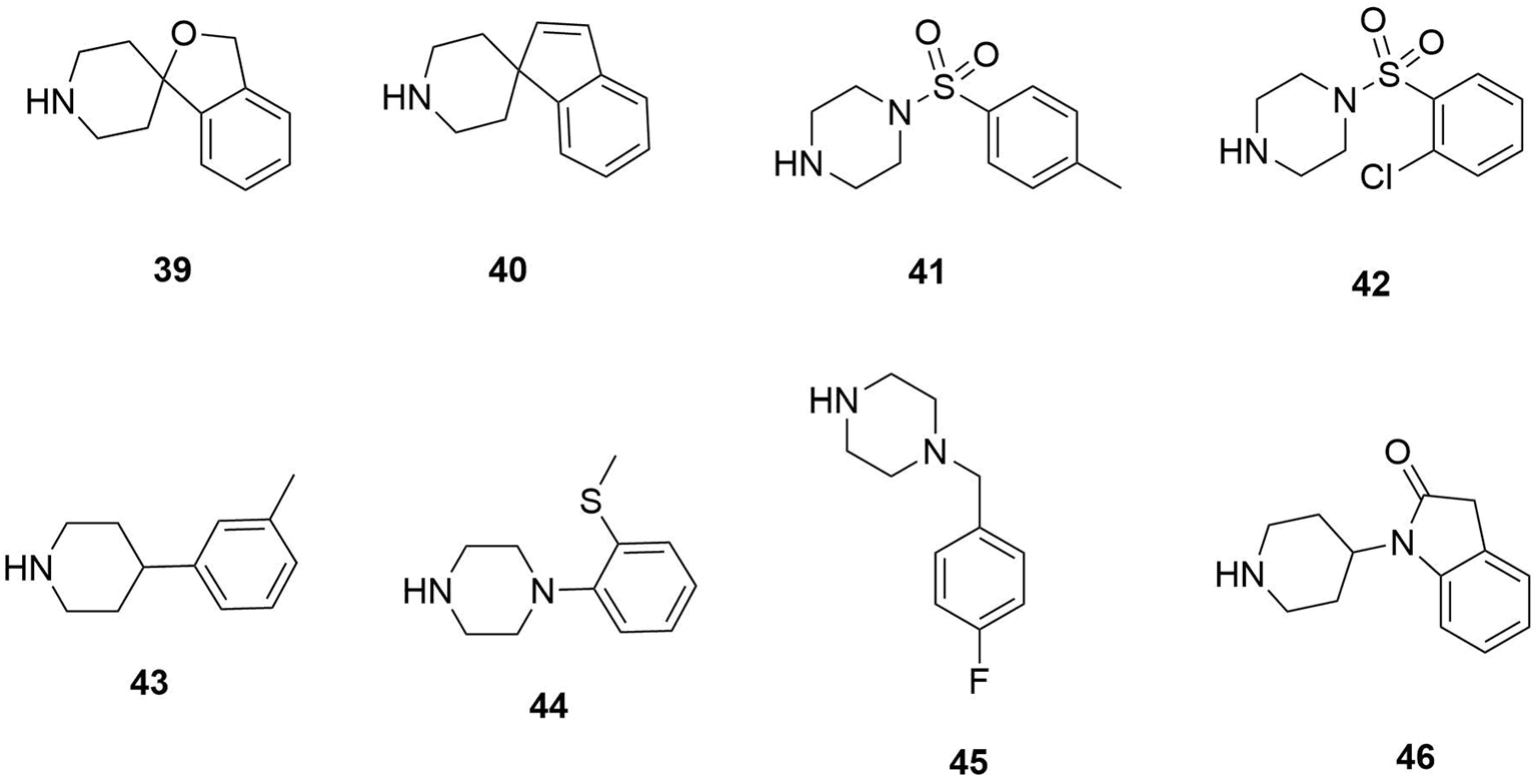
Study binding mode variations for the identified active fragment hits [46]

*Pf*DHFR is a wild-type and mutant receptor. This has also been explored by Hoarau et al. (2020), where the researchers used docking techniques to screen and identify new *Pf*DHFR inhibitors to act as antimalarial therapy [46]. In this experiment, molecular docking was used to analyze selected fragment hits where it complemented crystallographic data to study binding mode variations for the identified active fragment hits (**39**–**46**) (Fig. 10). [46]The findings of this research indicated that the fragment-based strategy was efficient and specific since the active fragment hits were highly selective for the target enzyme.

### Molecular dynamics

Molecular dynamics simulation, commonly referred as MD, is a computer simulation method that dates back to the eighteenth century and is used to analyze the physical movement of atoms and molecules in a mimicked physiological environment. This tool is important for understanding the physical basis of the structure and function of biological molecules. Optimization of the model allows the protein to assume its lowest energy conformation state, which renders it stable.

The stability of the protein is monitored by observing the fluctuations within the protein structure throughout the MD simulation until the system reaches a point of convergence. The Root Mean Square Deviation (RMSD) is used to measure the average change in displacement of a selection of atoms for a particular frame with respect to a reference frame. It is calculated for all frames in the trajectory. The values of the acceptable range of protein stability differ from system to system. Literature has, however, suggested that a low root mean squared deviation of approximately 2 to 3.5 Angstroms is an indication of an accurate model. [47]

### Virtual screening and pharmacophores

Virtual screening (VS) is a docking method that is used to search libraries of small molecules to identify ligands that are most likely to bind to a drug target, typically a protein receptor or enzyme. The mechanism of action of traditional antimalarial agents such as quinolones involves the interference of the digestion of haemoglobin during the blood stages of the malaria life cycle [48]. The new ligands being explored via VS show a different mechanism of action which is useful to overcome resistance. Although for some of these drugs the mechanism of action remains unknown, some are proposed to interfere with signal transduction pathways by inhibiting enzymes such as phosphatidylinositol 4-kinase (PI4K). Some are hypothesized to inhibit lipid storage and vesicular trafficking pathways [49].

Compared to traditional high-throughput screening, virtual screening is a somewhat more direct and rational approach to drug discovery as it has the advantage of low cost and, at the same time, a highly effective screening strategy. Successful hits from virtual screening can then lead to either the synthesis or purchase of these ligands, followed by biological evaluation to validate the results.

Proekt defined a pharmacophore as an ensemble of steric and electronic features necessary to ensure the optimal supramolecular interactions with a specific biological target and trigger (or block) its biological response [50]. This allows for the formation of a “super” ligand as certain functional groups can be selected and pieced together to form a ligand that is best suited to the receptor.

Pharmacophore modeling is one of the computer-aided drug design (CADD) methods utilized to address the expensive and complex drug discovery process. Generated pharmacophore models increase the understanding of ligand–protein interactions, with a pharmacophore defined as a molecular frame describing the vital features responsible for a molecule’s biological activity. In ligand-based pharmacophore modeling, the model can be built using the active ligand’s structural information in case the target structure is unavailable. Ligand-based approaches use a set of available active ligands to design novel ligands. This approach is utilized when the target protein's structure is unknown. The active ligands are identified first using the literature available or database search, where the data set is split into a training set and a test set. Feature analysis will then be done for the training set ligands and common features detected by aligning the active molecules. Subsequently, the pharmacophore model is then generated, and the developed models are ranked before validation and selection of the best pharmacophore model based on the results. Meanwhile, structure-based pharmacophore modeling uses the available structure of the target where the model is built using its structural properties. This is used when the structure of the target protein is known. The first step is selecting and preparing the target protein structure, followed by binding site prediction. A careful analysis will then identify the complemental chemical features of the binding site amino acids and their layouts before generating optimized pharmacophore features. The final step is selecting the crucial pharmacophore features responsible for the activity.

**Fig. 11:**
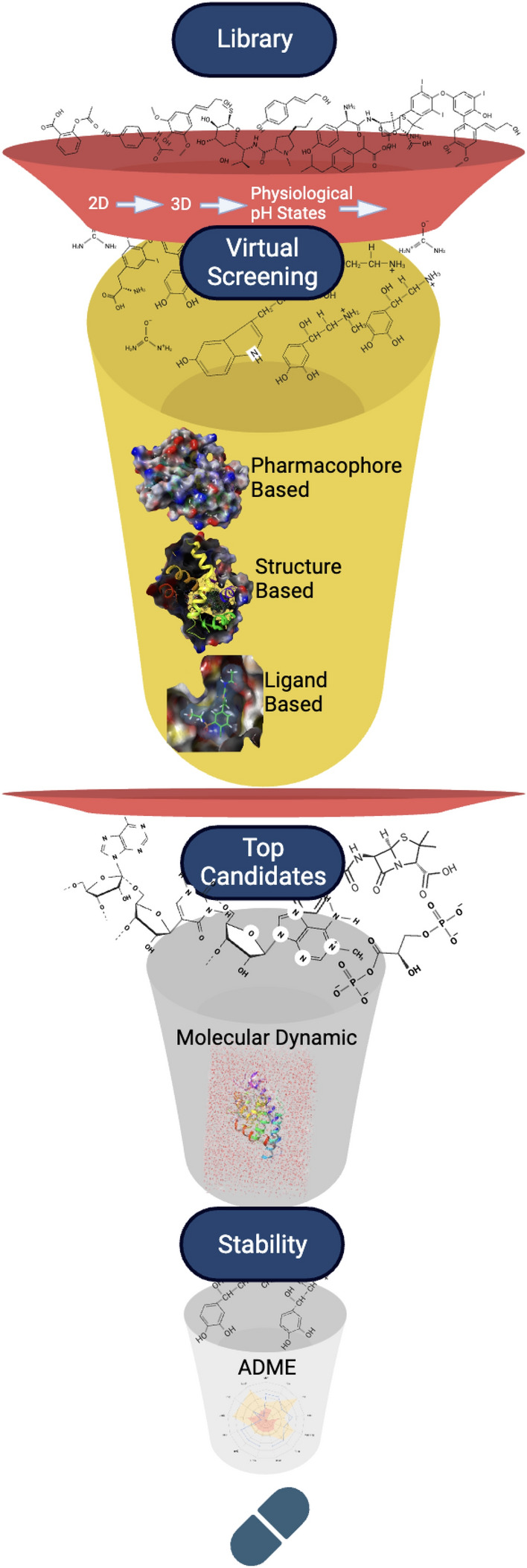
Virtual screening (VS) workflow for finding novel inhibitors

The existing pharmacophore modeling software are used for virtual screening (VS) (Fig. 11), drug target fishing, docking, ligand profiling, and ADMET prediction. Molecular pharmacophore patterns derived from converting each atom or group of a compound with features associated with molecular recognition can be hydrogen bond donors (HBD), aromatic rings, positively charged groups, negatively charged groups, hydrophobic groups, and their combinations.

Due to its simplicity and versatility, the pharmacophore modeling strategy is also considered for drug repurposing and predicting side effects. Its applications in VS entail identifying compounds that trigger the intended biological activity. Here, scientists generate a pharmacophore model that codes the 3D structure of the desired interaction pattern correctly. In cases where several active ligands and their inactive derivatives exist, validation entails separating the ligand data into training and test sets. The drug target fishing applications involve elucidating a drug molecule’s mechanism of action that is not fully understood. Similar ligands are identified using cheminformatics-based similarity search tools. The compound under investigation can also be the query to determine the most suitable pharmacophore model. At the same time, the method can also be applied to find a target for a molecule with unknown activity. Pharmacophore modeling used in ligand profiling further estimates the possible targets and their adverse effects and suggests new drug targets. Pharmacophore- and docking-based approaches are used for docking to generate better outcomes and overcome limitations. Pharmacophore models are applied as initial filters to reduce the number of molecules for docking, as guides during docking, and as filters after docking to select ligands and rank the poses. They are utilized as filters to determine molecules meeting basic structural and chemical functionality requirements before docking. In ADMET profiling, the model estimates the properties early to limit failures in novel drug development.

However, there are challenges in pharmacophore modeling, such as yielding inactive molecules due to a lack of good scoring functions when using VS by pharmacophore. The compounds that match the query may differ from other known inhibitors and contain functional groups that do not bind with the receptor binding site. Moreover, there is a chance for higher false positive rates with pharmacophore-VS approaches. A lack of the required hypothesis, pharmacophore quality, and discrepancies from real biological conditions cause this limitation. There is also a challenge of ligand flexibility because VS utilizes databases with low energy structures per molecule. Protein flexibility and ligand conformational flexibility are other limitations.

Computational methods such as VS have allowed for the discovery of new drugs such as cipargarmin, which has completed phase 2 of clinical trials. DSM625 is another example of a drug that was discovered among a series of novel triazolopyrimidine-based inhibitors and is also undergoing clinical trials [51].

## Conclusion

As the world grapples with the relentless challenges posed by diseases like malaria, the advent of sophisticated computational tools has emerged as a beacon of hope in the quest for effective treatments. These tools, encompassing virtual screening, molecular docking, artificial intelligence (AI), and machine learning (ML), have revolutionized the drug discovery landscape, empowering medicinal chemists to explore uncharted territories in the search for novel compounds and repurposing strategies. Virtual screening, a cornerstone of modern drug discovery, has transformed the way potential drug candidates are identified. By sifting through vast libraries of compounds, virtual screening algorithms can rapidly pinpoint molecules that exhibit promising interactions with target proteins, significantly streamlining the drug discovery process. Molecular docking, a computational technique that simulates the molecular interactions between a drug candidate and its target protein, has proven invaluable in developing antimalarial compounds. By elucidating the intricate binding modes of potential drugs, molecular docking has guided the synthesis of highly specific and potent antimalarials, bringing us closer to eradicating this age-old disease.

AI and ML, the driving forces behind the Fourth Industrial Revolution (4IR), have also significantly contributed to the fight against malaria. These powerful tools have enabled the analysis of vast genomic, proteomic, and clinical data datasets, revealing hidden patterns and correlations that inform drug target identification and compound optimization. QSAR modeling, a computational technique that links the structural features of compounds to their biological activities, has emerged as a valuable tool in antimalarial drug discovery. By establishing these relationships, QSAR models can predict the effectiveness of potential drug candidates, guiding medicinal chemists toward the most promising avenues for drug development. The convergence of these computational strategies, coupled with the ever-increasing power of computing systems, has ushered in a new era of drug discovery, holding immense promise for the eradication of malaria. As we continue to harness the power of these tools, we move closer to a future where malaria is no longer a threat, but a relic of the past.
